# Serum Matrix Metalloproteinase 9 and Macrophage Migration Inhibitory Factor (MIF) Are Increased in Young Healthy Nonobese Subjects with Positive Family History of Type 2 Diabetes

**DOI:** 10.1155/2018/3470412

**Published:** 2018-09-13

**Authors:** Agnieszka Nikołajuk, Natalia Matulewicz, Magdalena Stefanowicz, Monika Karczewska-Kupczewska

**Affiliations:** ^1^Department of Prophylaxis of Metabolic Diseases, Institute of Animal Reproduction and Food Research, Polish Academy of Sciences, Olsztyn, Poland; ^2^Department of Metabolic Diseases, Medical University of Białystok, Białystok, Poland; ^3^Department of Internal Medicine and Metabolic Disorders, Medical University of Białystok, Białystok, Poland

## Abstract

Insulin resistance increases the risk for cardiovascular disease (CVD) even in the absence of classic risk factors, such as hyperglycemia, hypertension, dyslipidemia, and obesity. Low-grade chronic inflammatory state is associated both with insulin resistance and atherosclerosis. An increased circulating level of proinflammatory proatherogenic factors and biomarkers of endothelial activation was observed in diabetes and CVD. The aim of our study was to assess serum proatherogenic and proinflammatory factors in young healthy nonobese subjects with positive family history of type 2 diabetes. We studied 74 young healthy nonobese subjects with normal glucose tolerance (age < 35 years, BMI < 30 kg/m^2^), 29 with positive family history of type 2 diabetes (relatives, 25 males and 4 females) and 45 subjects without family history of diabetes (control group, 39 males and 6 females). Hyperinsulinemic-euglycemic clamp was performed, and serum concentrations of monocyte chemoattractant protein-1 (MCP-1), interleukin 18 (IL-18), macrophage inhibitory cytokine 1 (MIC-1), macrophage migration inhibitory factor (MIF), matrix metalloproteinase (MMP-9), and soluble forms of adhesion molecules were measured. Relatives had markedly lower insulin sensitivity (*p* = 0.019) and higher serum MMP-9 (*p* < 0.001) and MIF (*p* = 0.006), but not other chemokines and biomarkers of endothelial function. Insulin sensitivity correlated negatively with serum MMP-9 (*r* = −0.23, *p* = 0.045). Our data show that young healthy subjects with positive family history of type 2 diabetes already demonstrate an increase in some nonclassical cardiovascular risk factors.

## 1. Introduction

Insulin resistance is a major risk factor for type 2 diabetes (T2D), atherosclerosis, and cardiovascular disease (CVD) [1]. It may be present in a significant part of apparently healthy, young subjects [2]. Insulin resistance increases the risk for CVD even in the absence of classic risk factors, such as hyperglycemia, hypertension, dyslipidemia, and obesity [3]. Chronic low-grade inflammation may be a link between insulin resistance, T2D, and atherosclerosis [4].

In insulin resistance, the development of atherosclerosis is associated with endothelial dysfunction, infiltration of immune cells into the arterial vessel wall, monocyte differentiation, and foam cell formation with degradation of the arterial extracellular matrix (ECM), migration, and proliferation of smooth muscle cells. These processes lead to the progression and manifestation of atherosclerotic lesion with subsequent plaque rupture and thrombosis. Proinflammatory cytokines and chemokines, secreted by inflammatory cells, may be key mediators of these processes. These factors play an important role in promoting the development of inflammation and are involved in all stages of atherosclerosis [1, 4, 5].

Monocyte chemoattractant protein-1 (MCP-1) is one of the important chemoattractant cytokines, which takes part in the process of recruitment and migration of monocytes into the arterial vessel wall and their differentiation into macrophages and lipid-loaded foam cells [6]. Another important proinflammatory cytokine is macrophage migration inhibitory factor (MIF), which displays chemokine-like functions and promotes arrest and chemotaxis of monocytes, neutrophils, and T cells in the area of inflammation [7, 8]. MIF might also contribute to cell recruitment by induction of MCP-1 [9] and other inflammatory mediators, such as adhesion molecules [10]. Inhibition of MIF signaling resulted in a significantly reduced number of macrophages in atherosclerotic plaques, suggesting a slower atheroprogression [11]. Both MCP-1 and MIF have also been implicated in the pathogenesis of insulin resistance [12, 13]. Serum concentrations of these factors, as well as other proinflammatory chemokines, like interleukin 18 (IL-18), are related to insulin action and are increased in T2D and CVD [14–17].

Pathologically accelerated subendothelial vascular remodeling in atherosclerotic process is related to ECM remodeling. The degradation of components of the ECM and regulation of arterial wall architecture are mainly mediated by the matrix metalloproteinase (MMP) family. MMP is synthesized by endothelial cells, neutrophils, systemic-circulatory monocytes, and local tissue macrophages [18]. MMP-9 is a key mediator of ECM remodeling, which has emerged as a biomarker of CVD [19, 20] and is also associated with insulin resistance [21] and T2D [22].

Adhesion of inflammatory cells to vascular endothelium is mediated by cell adhesion molecules, selectins and integrins [23]. Soluble forms of these molecules such as E-selectin (sE-selectin), intercellular adhesion molecule-1 (sICAM-1), and vascular cell adhesion molecule-1 (sVCAM-1) are released and may reflect overexpression of their respective membrane-bound forms [23]. Increased levels of these soluble forms are associated with insulin resistance, T2D, and CVD [24–26].

Most human studies suggested that low-grade chronic inflammation was a factor contributing to atherosclerosis and insulin resistance. However, these studies were mostly conducted in people with overt metabolic abnormalities, i.e., obesity, metabolic syndrome, T2D, and CVD. It is well known that central obesity, hyperglycemia, hypertriglyceridemia, low HDL cholesterol, and high blood pressure are risk factors for both CVD and T2D and may be confounding factors that affect the understanding relationships between inflammation and atherosclerosis. Thus, it remains unclear, what are the early biomarkers of inflammation and endothelial dysfunction, which are altered before the onset of T2D and CVD. It is relevant to study young, healthy subjects with different degrees of whole-body insulin sensitivity, but without overt metabolic disturbances. Offspring of T2D subjects demonstrate insulin resistance, and they are at increased risk of T2D because of their family history. Insulin resistance occurs before the onset of obesity and/or hyperglycemia in this group, which indicates a strong genetic background in the development of this phenomenon. The concordance rate for T2D in monozygotic twins is high; however, it is less than 100%, which indicates that environmental factors may also play a role [27, 28]. Therefore, the aim of the present study was to assess serum concentrations of proatherogenic and proinflammatory factors in young healthy subjects with positive family history of T2D.

## 2. Subjects and Methods

### 2.1. Study Participants

The study group consisted of 74 young (age < 35 years) healthy nonobese (body mass index (BMI) < 30 kg/m^2^) volunteers: 29 with positive family history of type 2 diabetes (relatives, 25 male and 4 female subjects) and 45 subjects without family history of diabetes (control group, 39 male and 6 female subjects). Relatives of type 2 diabetic patients were recruited for the study if both parents had type 2 diabetes or if one parent and one first- or second-degree relative had type 2 diabetes.

All study participants had no cardiovascular disease, hypertension, and hyperandrogenism or had no clinical and laboratory signs of inflammation and other serious medical problems; all were nonsmokers. Subjects were excluded if they had any inflammatory disease within the last 3 months. All subjects did not take any anti-inflammatory drugs and drugs known to affect glucose and lipid metabolism. Body weight of the subjects had remained stable for at least 3 months before the study. All analyses were performed after an overnight fast. A standard 75 g oral glucose tolerance test (OGTT) was performed, and all subjects had normal glucose tolerance according to the World Health Organization criteria. The study protocol was approved by the Ethical Committee of Human Studies of the Medical University of Bialystok, Poland. All participants gave written informed consent before entering the study. Before entering the study, a physical examination and appropriate laboratory tests were performed. Anthropometric measurements were performed as previously described [29].

### 2.2. Insulin Sensitivity

Insulin sensitivity was evaluated by the hyperinsulinemic-euglycemic clamp, as previously described [29]. The rate of the whole-body glucose uptake (*M* value) was calculated as the mean glucose infusion rate from 80 to 120 min, corrected for glucose space, and normalized per kilogram of fat-free mass (ffm).

### 2.3. Biochemical Analyses

Fasting blood samples were taken from the antecubital vein before the beginning of the clamp. Before the determination of serum insulin, proinflammatory cytokines, chemokines, and adhesion molecule concentrations, the samples were frozen at −80°C until the analyses were carried out. Plasma glucose was measured immediately by the enzymatic method using a glucose analyzer (YSI 2300 STAT Plus, Yellow Springs, OH, USA) during the OGTT and the clamp study. Serum total cholesterol, HDL cholesterol, triglycerides (TG), and LDL cholesterol were assayed enzymatically using a multiparametric analyzer (Cobas c111, Roche Diagnostics). Serum insulin was measured with immunoradiometric assay (IRMA), (DiaSource Europe, Nivelles, Belgium). Serum high-sensitivity C-reactive protein (hsCRP) was measured with particle-enhanced immunonephelometry (Dade Behring, Marburg, Germany).

Serum MIF, MCP-1, MMP-9, and macrophage inhibitory cytokine-1 (MIC-1) concentrations were measured with enzyme-linked immunosorbent assay kits from R&D Systems (Minneapolis, MN, USA). The sensitivity and intra-assay and interassay coefficients of variation (CVs) of these assays were 0.068 ng/ml, 9.7% and 7.9% for MIF; 1.7 pg/ml, 7.8% and 6.7% for MCP-1; 0.156 ng/ml, 9.7% and 7.9% for MMP-9; and 2.1 pg/ml, 2.5% and 6.1% for MIC-1. Serum levels of soluble adhesion molecules (sICAM-1, sVCAM-1, and sE-selectin) were measured with ELISA kits (R&D Systems Inc., Minneapolis, MN, USA). The minimum detectable concentration was 0.049 ng/ml for sICAM, 1.26 ng/ml for sVCAM-1, and 0.003 ng/ml for sE-selectin. The intra-assay and interassay CVs for sICAM were below 5 and 6.8%; for sVCAM, below 3.6 and 7.8%; and for sE-selectin, below 6.9 and 8.6%, respectively. Serum IL-18 was measured with an MBL enzyme-linked immunosorbent assay kit (Nagoya, Aichi, Japan) with sensitivity of the method and intra-assay and interassay CVs: 12.5 pg/ml, 10.8% and 10.1%, respectively.

### 2.4. Statistical Analysis

The statistics were performed with STATISTICA 10.0 software. The differences between the groups were estimated with unpaired Student's *t*-test. The relationships between variables were studied with the Pearson product-moment correlation analysis. The level of significance was accepted at *p* < 0.05.

## 3. Results

The clinical characteristics of the study groups are shown in Table 1. The subjects with a family history of type 2 diabetes did not differ significantly in the anthropometric parameters from the control group. Serum glucose concentrations and the lipid profile were similar in both groups. Fasting insulin serum concentrations were significantly higher in the offspring of type 2 diabetic subjects (*p* = 0.027). Additionally, relatives had markedly lower insulin sensitivity (*p* = 0.019).

**Figure 1:**
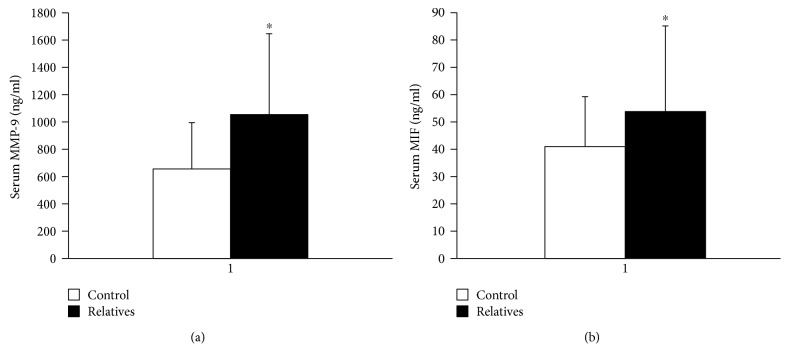
Serum MMP-9 (a) and MIF (b) concentrations in the study groups.

**Figure 2:**
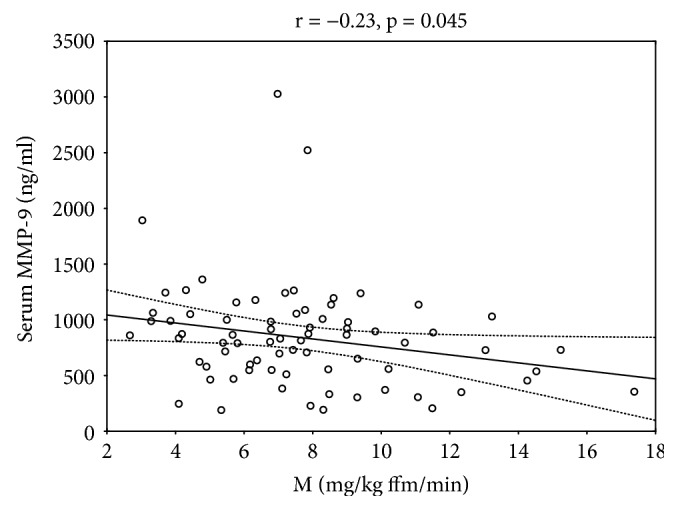
Correlation between serum MMP-9 and insulin sensitivity.

Serum hsCRP, IL-18, MCP-1, MIC-1, sVAM-1, sICAM-1, and sE-selectin did not differ significantly between the groups. We observed an increase in serum MMP-9 (Figure 1(a)) and MIF (Figure 1(b)) in the relative group in comparison to the control group (*p* < 0.001 and *p* = 0.006, respectively). Serum MMP-9 level was weakly associated with insulin sensitivity (*r* = −0.23, *p* = 0.045) (Figure 2).

## 4. Discussion

The main finding of the present study is an increase in serum MMP-9 and MIF concentrations in healthy nonobese subjects with family history of type 2 diabetes. MMP-9 was inversely related to insulin sensitivity.

The elevated levels of circulating proinflammatory biomarkers have been suggested to predict cardiovascular disease. It might be the primary abnormality preceding the atherosclerotic process in individuals at high risk of type 2 diabetes. Insulin resistance is an important pathophysiological factor contributing to the development of CVD [2–4].

MMP-9 contributes to the arterial vessel wall remodeling in atherosclerotic process. It directly degrades ECM proteins and activates cytokines and chemokines [19]. Roberts et al. found elevated levels of MMP-9 in men with metabolic syndrome and observed that they displayed significant reductions in BMI, insulin, HOMA-IR, and MMP-9 after diet and exercise intervention [30]. In the study of Yu et al., subjects which exhibited at least one of the features of metabolic syndrome (i.e., central obesity, low HDL cholesterol, hypertension, elevated fasting blood glucose, and high TG) had also higher serum MMP-9 comparing to subjects without features of metabolic syndrome [31]. MMP-9 plasma concentrations were also elevated in obese children with and without hypertension [32]. In our study, MMP-9 was negatively related to insulin sensitivity. This correlation was weak probably because of only relatively mild degree of insulin resistance in the T2D relative group. Importantly, our study subjects did not have obesity and any features of metabolic syndrome which could influence serum MMP-9 concentrations. Our data indicate that an increase in serum MMP-9 may precede the development of obesity and disturbances of glucose tolerance and that the offspring of type 2 diabetic patients are at elevated risk for CVD. Insulin resistance may be associated with the pathological subendothelial vascular remodeling.

We also found an increase in serum MIF concentrations in subjects with family history of T2D. As already mentioned, increased serum MIF concentration in impaired glucose tolerance and T2D was reported [15]. MIF gene variants were associated with the risk of T2D [33]. However, serum MIF concentrations were associated with T2D risk only in women and not in men and this association was stronger in obese than nonobese women [33]. Our study population was nonobese and consisted mostly of men, indicating that MIF may be associated with the predisposition to T2D even in the absence of obesity. In experimental studies, MIF deficiency partially protected from the development of insulin resistance, adipose tissue inflammation, and atherosclerosis in LDL-receptor-deficient [13] and high-fat-fed mice [34]. In our study, we did not observe a relationship between the concentration of MIF and insulin resistance. However, it should be noted that the group with family history of T2D in our study exhibited only a relatively mild degree of insulin resistance, which makes the potential relationships more difficult to detect. Our data support the results about the association between MIF and the predisposition to T2D. Given the aforementioned MIF proatherogenic actions [7–11], higher serum MIF concentration in the group with family history of T2D provides further evidence about an increased CVD risk in the offspring of T2D subjects.

Serum MCP-1 concentration did not differ between the study groups. As already mentioned, MCP-1 is involved in the development of atherosclerosis [6] and may also contribute to the development of insulin resistance [12]. In the study of middle-aged overweight and obese individuals, including subjects with an impaired glucose tolerance and type 2 diabetes, serum MCP-1 was positively related to fasting and postload glucose and TG and inversely to HDL cholesterol and QUICKI, an index of insulin sensitivity [14]. MCP-1G-2518 gene variant was inversely related to plasma MCP-1 values and the prevalence of insulin resistance and T2D [35]. However, in another study, genotype frequencies were similar in diabetic and nondiabetic subjects and were not related to serum MCP-1 [36]. Our data do not indicate MCP-1 as a primary factor contributing to insulin resistance in subjects at risk of developing type 2 diabetes. Indeed, a recent study demonstrated that insulin resistance preceded an increased MCP-1 production [37]. MCP-1 may also be induced by oxidative stress and other factors [38].

We did not find elevated levels of sICAM-1, sVCAM-1, and sE-selectin in the subjects with positive family history of type 2 diabetes. Caballero et al. reported an increased serum sVCAM concentrations in the offspring of T2D patients and elevated levels of sICAM in subjects with IGT or T2D [39]. However, lack of an increase in circulating soluble forms of adhesion molecules was also reported by other researchers [40]. Increased serum sE-selectin in subjects with T2D but not in their relatives was reported [41]. It was demonstrated that only sE-selectin was related to insulin resistance, whereas sICAM and sVCAM were associated with hyperglycemia rather than hyperinsulinemia or insulin resistance [42]. In the study of relatives of subjects with T2D with normal and impaired glucose tolerance [40], serum adhesion molecule concentration, although not increased, was related to inflammatory factors. Our data, together with the aforementioned studies, suggest that an increase in markers of endothelial dysfunction is rather secondary to inflammation, insulin resistance, and associated factors.

Our data suggest that the relatives of T2D subjects are at increased risk of CVD. Interestingly, we did not observe an increase in the concentrations of all the markers examined, but specifically MMP-9 and MIF. Given the fact that we studied generally healthy population, without other CVD risk factors, younger than in other studies [43, 44], our data indicate what may be the primary abnormalities leading to an increased CVD risk in subjects predisposed to develop T2D. However, our data do not reveal cause-effect relationships between analyzed serum biomarkers and insulin resistance. We only demonstrated that both insulin resistance and an increase in serum MMP-9 and MIF coexist in the group of subjects with family history of T2D. In our recent study, performed in a group of young and healthy subjects, we observed that inflammatory parameters are not the primary factors which associate with the development of insulin resistance [45]. However, adipose tissue MMP-9 expression, as a marker of ECM remodeling, but not serum MMP-9 concentration, was independently associated with insulin sensitivity [44]. Our data do not reveal what is the source of increased circulating MMP-9 and MIF concentrations in relatives of subjects with T2D.

## 5. Conclusions

In conclusion, our data show that young healthy subjects with positive family history of type 2 diabetes already demonstrate an increase in some nonclassical cardiovascular risk factors.

## Figures and Tables

**Table 1:** Clinical and biochemical characteristics of the study groups.

	Control (*n* = 45)	Family history of type 2 diabetes (*n* = 29)
Age (years)	22.96 ± 2.36	22.79 ± 2.21
BMI (kg/m^2^)	23.89 ± 2.70	24.33 ± 2.87
Waist (cm)	84.35 ± 8.16	85.76 ± 8.95
% body fat	20.76 ± 7.42	18.98 ± 7.73
Fasting glucose (mg/dl)	86.29 ± 8.04	87.19 ± 9.33
Fasting insulin (*μ*IU/ml)	10.03 ± 4.32	12.60 ± 8.31^∗^
*M* (mg/kg_ffm_/min)	7.76 ± 3.02	6.36 ± 1.83^∗^
Cholesterol (mg/dl)	167.58 ± 31.10	170.13 ± 33.78
TG (mg/dl)	79.97 ± 38.32	96.41 ± 50.77
HDL cholesterol (mg/dl)	61.73 ± 11.55	67.53 ± 12.68
LDL cholesterol (mg/dl)	96.59 ± 31.13	101.27 ± 35.82
hsCRP (mg/l)	0.56 ± 0.49	0.50 ± 0.47
IL-18 (pg/ml)	247.73 ± 84.18	240.64 ± 58.60
MCP-1 (pg/ml)	356.59 ± 141.85	370.88 ± 151.35
MIC-1 (pg/ml)	365.77 ± 112.90	356.31 ± 113.36
sE-selectin (ng/ml)	34.25 ± 14.73	36.62 ± 14.11
sVCAM (ng/ml)	792.09 ± 167.12	732.64 ± 173.61
sICAM (ng/ml)	230.30 ± 46.51	226.55 ± 41.83

Data are presented as mean ± SD; ^∗^*p* < 0.05.

## Data Availability

The data used to support the findings of this study are available from the corresponding author upon request.

## References

[1] DeFronzo R. A. (2010). Insulin resistance, lipotoxicity, type 2 diabetes and atherosclerosis: the missing links. The Claude Bernard Lecture 2009. *Diabetologia*.

[2] Eckel R. H., Grundy S. M., Zimmet P. Z. (2005). The metabolic syndrome. *Lancet*.

[3] Bonora E., Kiechl S., Willeit J. (2007). Insulin resistance as estimated by homeostasis model assessment predicts incident symptomatic cardiovascular disease in Caucasian subjects from the general population: the Bruneck study. *Diabetes Care*.

[4] Fernandez-Real J. M., Ricart W. (2003). Insulin resistance and chronic cardiovascular inflammatory syndrome. *Endocrine Reviews*.

[5] Deng X. L., Liu Z., Wang C., Li Y., Cai Z. (2017). Insulin resistance in ischemic stroke. *Metabolic Brain Disease*.

[6] Gu L., Okada Y., Clinton S. K. (1998). Absence of monocyte chemoattractant protein-1 reduces atherosclerosis in low density lipoprotein receptor-deficient mice. *Molecular Cell*.

[7] Bernhagen J., Krohn R., Lue H. (2007). MIF is a noncognate ligand of CXC chemokine receptors in inflammatory and atherogenic cell recruitment. *Nature Medicine*.

[8] Zernecke A., Bot I., Djalali-Talab Y. (2008). Protective role of CXC receptor 4/CXC ligand 12 unveils the importance of neutrophils in atherosclerosis. *Circulation Research*.

[9] Gregory J. L., Morand E. F., McKeown S. J. (2006). Macrophage migration inhibitory factor induces macrophage recruitment via CC chemokine ligand 2. *The Journal of Immunology*.

[10] Cheng Q., McKeown S. J., Santos L. (2010). Macrophage migration inhibitory factor increases leukocyte-endothelial interactions in human endothelial cells via promotion of expression of adhesion molecules. *The Journal of Immunology*.

[11] Müller I. I., Chatterjee M., Schneider M. (2014). Gremlin-1 inhibits macrophage migration inhibitory factor-dependent monocyte function and survival. *International Journal of Cardiology*.

[12] Kanda H., Tateya S., Tamori Y. (2006). MCP-1 contributes to macrophage infiltration into adipose tissue, insulin resistance, and hepatic steatosis in obesity. *The Journal of Clinical Investigation*.

[13] Verschuren L., Kooistra T., Bernhagen J. (2009). MIF deficiency reduces chronic inflammation in white adipose tissue and impairs the development of insulin resistance, glucose intolerance, and associated atherosclerotic disease. *Circulation Research*.

[14] Piemonti L., Calori G., Lattuada G. (2009). Association between plasma monocyte chemoattractant protein-1 concentration and cardiovascular disease mortality in middle-aged diabetic and nondiabetic individuals. *Diabetes Care*.

[15] Herder C., Kolb H., Koenig W. (2006). Association of systemic concentrations of macrophage migration inhibitory factor with impaired glucose tolerance and type 2 diabetes: results from the cooperative health research in the region of Augsburg, survey 4 (KORA S4). *Diabetes Care*.

[16] Makino A., Nakamura T., Hirano M. (2010). High plasma levels of macrophage migration inhibitory factor are associated with adverse long-term outcome in patients with stable coronary artery disease and impaired glucose tolerance or type 2 diabetes mellitus. *Atherosclerosis*.

[17] Blankenberg S., Luc G., Ducimetière P. (2003). Interleukin-18 and the risk of coronary heart disease in European men. The prospective epidemiological study of myocardial infarction (PRIME). *Circulation*.

[18] Galis Z. S., Khatri J. J. (2002). Matrix metalloproteinases in vascular remodeling and atherogenesis: the good, the bad, and the ugly. *Circulation Research*.

[19] Blankenberg S., Rupprecht H. J., Poirier O. (2003). Plasma concentrations and genetic variation of matrix metalloproteinase 9 and prognosis of patients with cardiovascular disease. *Circulation*.

[20] Kobayashi N., Hata N., Kume N. (2011). Matrix metalloproteinase-9 for the earliest stage acute coronary syndrome. *Circulation Journal*.

[21] Unal R., Yao-Borengasser A., Varma V. (2010). Matrix metalloproteinase-9 is increased in obese subjects and decreases in response to pioglitazone. *The Journal of Clinical Endocrinology and Metabolism*.

[22] Signorelli S. S., Malaponte G., Libra M. (2005). Plasma levels and zymographic activities of matrix metalloproteinases 2 and 9 in type II diabetics with peripheral arterial disease. *Vascular Medicine*.

[23] Libby P., Ridker P. M., Maseri A. (2002). Inflammation and atherosclerosis. *Circulation*.

[24] El-Mesallamy H. O., Hamdy N. M., Salman T. M., Ibrahim S. M. (2012). Adiponectin and sE-selectin concentrations in relation to inflammation in obese type 2 diabetic patients with coronary heart disease. *Angiology*.

[25] Matsumoto K., Sera Y., Nakamura H., Ueki Y., Miyake S. (2002). Serum concentrations of soluble adhesion molecules are related to degree of hyperglycemia and insulin resistance in patients with type 2 diabetes mellitus. *Diabetes Research and Clinical Practice*.

[26] Blankenberg S., Rupprecht H. J., Bickel C. (2001). Circulating cell adhesion molecules and death in patients with coronary artery disease. *Circulation*.

[27] Lattuada G., Sereni L. P., Ruggieri D. (2004). Postabsorptive and insulin-stimulated energy homeostasis and leucine turnover in offspring of type 2 diabetic patients. *Diabetes Care*.

[28] Willemsen G., Ward K. J., Bell C. G. (2015). The concordance and heritability of type 2 diabetes in 34,166 twin pairs from international twin registers: the discordant twin (DISCOTWIN) consortium. *Twin Research and Human Genetics*.

[29] Karczewska-Kupczewska M., Kowalska I., Nikolajuk A. (2012). Circulating brain-derived neurotrophic factor concentration is downregulated by intralipid/heparin infusion or high-fat meal in young healthy male subjects. *Diabetes Care*.

[30] Roberts C. K., Won D., Pruthi S. (2006). Effect of a short-term diet and exercise intervention on oxidative stress, inflammation, MMP-9, and monocyte chemotactic activity in men with metabolic syndrome factors. *Journal of Applied Physiology*.

[31] Yu A. P., Tam B. T., Yau W. Y. (2015). Association of endothelin-1 and matrix metallopeptidase-9 with metabolic syndrome in middle-aged and older adults. *Diabetology and Metabolic Syndrome*.

[32] Głowińska-Olszewska B., Urban M. (2007). Elevated matrix metalloproteinase 9 and tissue inhibitor of metalloproteinase 1 in obese children and adolescents. *Metabolism*.

[33] Herder C., Klopp N., Baumert J. (2008). Effect of macrophage migration inhibitory factor (MIF) gene variants and MIF serum concentrations on the risk of type 2 diabetes: results from the MONICA/KORA Augsburg case-cohort study, 1984-2002. *Diabetologia*.

[34] Finucane O. M., Reynolds C. M., McGillicuddy F. C. (2014). Macrophage migration inhibitory factor deficiency ameliorates high-fat diet induced insulin resistance in mice with reduced adipose inflammation and hepatic steatosis. *PLoS One*.

[35] Simeoni E., Hoffmann M. M., Winkelmann B. R. (2004). Association between the *A-2518G* polymorphism in the monocyte chemoattractant protein-1 gene and insulin resistance and type 2 diabetes mellitus. *Diabetologia*.

[36] Zietz B., Büchler C., Herfarth H. (2005). Caucasian patients with type 2 diabetes mellitus have elevated levels of monocyte chemoattractant protein-1 that are not influenced by the −2518 A→G promoter polymorphism. *Diabetes, Obesity and Metabolism*.

[37] Shimobayashi M., Albert V., Woelnerhanssen B. (2018). Insulin resistance causes inflammation in adipose tissue. *The Journal of Clinical Investigation*.

[38] Kumar A., Shalmanova L., Hammad A., Christmas S. E. (2016). Induction of IL-8 (CXCL8) and MCP-1 (CCL2) with oxidative stress and its inhibition with N-acetyl cysteine (NAC) in cell culture model using HK-2 cell. *Transplant Immunology*.

[39] Caballero A. E., Arora S., Saouaf R. (1999). Microvascular and macrovascular reactivity is reduced in subjects at risk for type 2 diabetes. *Diabetes*.

[40] Ruotsalainen E., Vauhkonen I., Salmenniemi U. (2008). Markers of endothelial dysfunction and low-grade inflammation are associated in the offspring of type 2 diabetic subjects. *Atherosclerosis*.

[41] Bannan S., Mansfield M. W., Grant P. J. (1998). Soluble vascular cell adhesion molecule-1 and E-selectin levels in relation to vascular risk factors and to E-selectin genotype in the first degree relatives of NIDDM patients and in NIDDM patients. *Diabetologia*.

[42] Blüher M., Unger R., Rassoul F., Richter V., Paschke R. (2002). Relation between glycaemic control, hyperinsulinaemia and plasma concentrations of soluble adhesion molecules in patients with impaired glucose tolerance or type II diabetes. *Diabetologia*.

[43] Ruotsalainen E., Salmenniemi U., Vauhkonen I. (2006). Changes in inflammatory cytokines are related to impaired glucose tolerance in offspring of type 2 diabetic subjects. *Diabetes Care*.

[44] Li M., Feng D., Zhang K., Gao S., Lu J. (2016). Disproportionately elevated proinsulin levels as an early indicator of *β*-cell dysfunction in nondiabetic offspring of Chinese diabetic patients. *International Journal of Endocrinology*.

[45] Matulewicz N., Stefanowicz M., Nikolajuk A., Karczewska-Kupczewska M. (2017). Markers of adipogenesis, but not inflammation, in adipose tissue are independently related to insulin sensitivity. *The Journal of Clinical Endocrinology and Metabolism*.

